# Lignin-Based Gel Polymer Electrolyte for Cationic Conductivity

**DOI:** 10.3390/polym13142306

**Published:** 2021-07-14

**Authors:** Nabi S. Shabanov, Kamil Sh. Rabadanov, Malik M. Gafurov, Abdulgalim B. Isaev, Dinara S. Sobola, Sagim I. Suleimanov, Akhmed M. Amirov, Abil Sh. Asvarov

**Affiliations:** 1 Analytical Center for Collective Use, Dagestan Federal Research Centre of the Russian Academy of Sciences, 367001 Makhachkala, Russia; shabanov.nabi@yandex.ru (N.S.S.); rksh@mail.ru (K.S.R.); malik52@mail.ru (M.M.G.); s.sagim.i@yandex.ru (S.I.S.); aamirov@mail.ru (A.M.A.); 2 Department of Inorganic Chemistry and Chemical Ecology, Dagestan State University, Ulitsa Batyraya 4a, 367008 Makhachkala, Russia; abdul-77@yandex.ru; 3 Department of Physics, Faculty of Electrical Engineering and Communication, Brno University of Technology, Technická 2848/8, 616 00 Brno, Czech Republic; 4 Institute of Physics of Materials, Academy of Sciences CR, Zizkova 22, 616 62 Brno, Czech Republic; 5 Shubnikov Institute of Crystallography, Federal Scientific Research Center, “Crystallography and Photonics” of Russian Academy of Sciences, 59, Leninsky Pr., 117333 Moscow, Russia; abil-as@list.ru

**Keywords:** gel polymer electrolytes, bio-sourced materials, lithium cation transfer

## Abstract

The article presents the results of the preparation and study of a gel-polymer electrolyte based on lignin obtained from *Pinus sylvestris*. Sulfonation and subsequent chlorination of lignin make possible implementation of the principle of mono-ionic conductivity in a natural biopolymer matrix, which provides predominantly cationic conductivity of the electrolyte. Based on the results of the qualitative and quantitative analysis of the synthesized samples, the mechanisms of the chemical conversion of the biopolymer, the structure models of the converted fragments of macromolecules, as well as the quantum-chemical calculation of their electronic and geometric parameters are presented. The key electronic characteristics of the gel polymer electrolytes (GPE) based on a composite of lignins with 20 wt.% polyvinyl alcohol are determined by impedance spectroscopy. The maximum value of the specific volume conductivity is 2.48 × 10^−4^ S cm^−1^, which is comparable with most commercial electrolytes of this type, but at the same time, record values are reached in the number of lithium cation transfer $t_{Li}^{+}$ of 0.89. The studies allow to identify the basic laws of the effect of chemical modification on the structure of GPE and describe the mechanism of ionic conductivity.

## 1. Introduction

In recent decades, there has been a noticeable interest in clean energy storage systems to meet the growing abundance of energy from intermittent renewable sources such as wind, sun and sea waves. The rechargeable lithium-ion battery (LIB) is considered to be one of the main energy storage devices enabling renormalization of said intermittent sources, due to its high output voltage, large specific capacity, and longer service life as compared to alternative devices [1,2,3]. The use of a liquid electrolyte in storage devices suffers from several drawbacks, including solvent leakage, risk of fire, and explosion. To solve these problems and increase the stability of LIBs, solid and pseudo-liquid electrolytes, such as polymer electrolyte (PE) [4,5,6] and gel polymer electrolyte (GPE) [7,8,9] were created.

The development of an electrolyte based on lignin (L), which is the second-most abundant biopolymer on Earth (after alginate) [10], would decrease the impact on the environment and provide devices for energy storage and conversion with cheap and readily available raw sources. L is a three-dimensional polymer consisting of nine-carbon phenolpropane units linked by various types of bonds. As a natural biopolymer, L is also more preferable for use than synthetic materials due to its higher biocompatibility. Moreover, L as a by-product of large-tonnage cellulose pulp production processes is currently readily available, cheap, and represents an under-utilized raw material [11]. About 50 million tons of L are produced annually worldwide, but less than 10% is used rather than burnt as low-grade biomass [12]. Therefore, extensive research aimed at finding sustainable uses of lignin is an extremely relevant research direction. The original natural biopolymer is inert from a chemical and physical (electronic) point of view, and it is suitable primarily as a fuel in unmodified form. At the same time, lignin is an inexhaustible source of cheap hydrocarbon, the processing and modification of which opens up a wide range of its applications. The main direction in solving this problem is modification of L by its chemical or fermentative transformation in order to change its physical and chemical properties [13,14,15]. There are very few publications where L is studied as an electrolyte. Papers [16,17,18,19] present the results of preparation of polyelectrolyte complexes, GPE, and aqueous electrolytes based on L. However, in currently available works, the possibility of using L as a macromolecular anion, i.e., the implementation of the principle of mono-ionic conductivity, is lacking or has not been studied in detail. Polymers with fixed acid functional groups in the matrix have several advantages associated with the stability of the LIB [20]. In particular, the cation is, as a rule, less mobile than the anion since their motion is strongly connected with the motion of the main Lewis sites in the polymer matrix. This is the reason for the small number of cation transfers with bipolar charge carriers in polymer electrolytes, which is usually less than 0.5. Besides, anions tend to accumulate on the anode and block it, forming a concentration gradient [21]. This leads to electrical polarization and, consequently, to a poor behavior, such as voltage loss, higher internal impedance and undesirable reactions, and higher likelihood of element failure [22]. Accordingly, the ultimate challenge facing green and sustainable energy approach is to achieve a balance between cost savings, superior operating performance of the device and use of environmentally friendly materials. Preparation of L-based electrolytes with embedded acid functional groups can significantly reduce manufacturing costs while maintaining the quality suitable for actual use in the LIB, which is an urgent task from both technological and economic points of view.

## 2. Materials and Methods

The L was prepared from coniferous wood of *Pinus sylvestris* by hydrolysis of its cellulose component. Lithium hydroxide (99.5%), sodium hydroxide (99.5%), sulfuric acid (93%), polyvinyl alcohol (6000) (Prime chemicals group, Moscow, Russia), sodium sulfide (99.5%) (JSC L.Ya. Karpov Chemical Plant, Kazan, Russia), and chlorine gas (99.6%) (M-gas Company, Moscow, Russia) were used.

### 2.1. Lignin Preparation

To obtain L, we used coniferous wood chips, from which L was isolated by acid treatment. The volume of 250 mL of 72% H_2_SO_4_ was added to 50 g of pulverized wood chips and kept at 24–25 °C for 2.5 h with intermittent gentle stirring. The mixture was then transferred into a 2000 mL conical flask and 1000 mL of distilled water was added. The diluted mixture was refluxed on electric stove for 1 h, allowing the formed the L particles to become larger and settle. Then the L was filtered, washed with deionized water, and dried at 110 °C for 3 h. The L yield achieved, defined as the ratio of the obtained mass of lignin to the mass of the original chips, was 40%.

To obtain a homogeneous mass, the precipitated L was hydrolyzed in an aqueous alkaline medium of 10% NaOH at 180 °C for 2 h. The process took place in a Teflon^®^-lined autoclave. As a result, low-molecular-weight water-soluble L was obtained, subsequently precipitated by neutralization with sulfuric acid. The precipitate was a brown homogeneous powder of the low-molecular-weight L. The resulting powder was also filtered, washed with deionized water, and dried.

### 2.2. Lignin Sulfonation

L was sulphonated by boiling in the autoclave in sodium sulfite solution of 10% Na_2_SO_3_. The volume of solution per gram of L was 10 mL. The boiling process was carried out in two stages. The first stage lasted for 1–1.5 h at 105–110 °C. This stage involved L swelling and impregnation with the salt system accompanied by partial sulfonation of the same L. The second stage lasted for 2–2.5 h at 160–180 °C and involved additional sulfonation of the L. It resulted in water-dissolved sodium lignosulfonate, which was converted into precipitate of lignosulfonic (LS) acid by acidification with sulfuric acid, and the obtained precipitate was then filtered, washed, and dried.

### 2.3. Chlorination of Lignosulfonic Acid

The obtained LS acid was additionally chlorinated. The 30 mL of deionized water was poured into 100 mL of flat-bottomed flask and then 3 g of the L was added. Chlorine gas was passed through the resulting mixture at the rate of 10 mL per minute for 3 h under vigorous stirring. During chlorination, the LS precipitate changed its color from dark brown to yellow. The resulting chloro-lignosulfonic acid (LS-Cl) was also filtered and washed with deionized water and ethanol.

### 2.4. Obtaining A Gel Polymer Electrolyte

The stages described above resulted in preparation of powders of low-molecular-weight L, LS acid, and LS-Cl acid, from which GPEs were formed. At the first stage, the powders were transferred to a soluble state by neutralizing L acid groups with lithium hydroxide. For this purpose, 10 mL of deionized water was added to 1 g of L and, under vigorous stirring, titrated with 0.1 M of LiOH under the control of a pH meter. For a complete dissolution of the L, titration was conducted up to pH = 8–9. The resulting solution was allowed to stand for 30 min. If pH value was less than 8, the solution was basified until stable values were established. The amount of LiOH consumed to neutralize completely its acid groups was calculated according to the formula:

(1) $$\nu_{Li}=\frac{C_{1}\times V_{1}-\left(10^{-\mathrm{pOH}}-10^{-7}\right)\times V_{2}}{m}$$

where *C*_1_ *V*_1_ are the titrant concentration (mol_Li_/g_lignin_) and volume, respectively, *V*_2_ is the volume of the solution to be titrated, pOH is the hydroxide value after titration, and *m* is the mass of the L quantity.

Then, in order to get rid of unreacted lithium hydroxide and, possibly, mineral salts formed, the solutions were treated with an ion-exchange resin until *pH* ≈ 7. As a result, solutions of lithium salts L, LS, and LS-Cl were obtained, which were then evaporated and dried at 80 °C in a vacuum oven. Next, membranes were formed from the obtained L salts. However, the main drawback of L is its relatively weak mechanical strength, which cannot meet the requirements of actual application in LIB [23]. Polyvinyl alcohol (PVA), which has excellent thermal, mechanical, and chemical stability and high film-forming properties was used to impart the required mechanical properties [24,25,26]. For this purpose, L: PVA, LS: PVA, and LS-Cl: PVA composites at the weight ratio of 80:20 were prepared by dissolving the corresponding polymers in dimethyl sulfoxide (DMSO). The obtained solutions with 20% concentration of polymers in DMSO were poured into Teflon^®^ dishes and dried in a dry box at 60 °C for 24 h. As a result, we obtained GPEs based on low-molecular-weight L (GPE-L), lignosulfonate (GPE-LS), and chloro-lignosulfonate (GPE-LS-Cl) in the form of flexible and elastic films (shown in Figure S1).

### 2.5. Characterization and Performance Evaluation

IR absorption spectra were recorded by the FT-IR spectrometer Vertex 70 (Bruker, Billerica, MA, USA) with the resolution of 2 cm^−1^ and a sample averaging of 128. Samples for studies were prepared from the mixture of KBr powder and lignin under study at the ratio of 250 mg/1 mg in the shape of pressed tablets. For all the spectra, the baseline was a polyline going through points with minimal absorption near wave numbers 740, 1850, and 3750 cm^−1^. Then, the corrected spectroscopic data were normalized to integral absorption in the considered spectral range of 740–1850.

Elemental analysis was performed by ASPEX Express electron microscope equipped by the OmegaMax EDX detector (ASPEX Corporation, Delmont, PA, USA) at accelerating voltage 20 kV.

Quantum-chemical calculations were performed using the HyperChem program by PM3 semi-empirical method. A distinctive feature of the PM3 method is that it satisfactorily reproduces structure and energy of the compounds. For all the compounds under study, the lowest spin multiplicity was specified. The spin state was calculated by the restricted Hartree-Fock method. To increase the convergence rate of calculations, the Accelerate convergence procedure (direct inversion of the iterative subspace) was used.

Thermal properties of the GPE were studied using an STA 449 F3 Jupiter simultaneous thermal analyzer from NETZSCH (Bobingen, Germany), at the heating rate of 5 °C/min in the temperature range of 30–350 °C. The sample mass was 9–10 mg.

The ionic conductivity of electrolytes was determined by impedance spectroscopy on a RLC meter E7-20 (St. Petersburg, Russia) in the frequency range from 25 Hz to 1 MHz and the signal amplitude of 100 mV within the temperature range of 20–50 °C. The cell was a cylindrical vessel with the diameter of 25 mm with a clamping electrode fixed on the pressure cap. The cell temperature was maintained by an RH25-6A water thermostat. The measured sample was sandwiched between two lithium electrodes with the diameter of ≈10 mm. The cell was prepared in a dry box in an inert atmosphere of argon (shown in Figure S2).

The values of the diffusion coefficient (*D*), mobility (*μ*), and the concentration of mobile charge carriers (*n*) were calculated according to the equations [27,28].

The values of the diffusion coefficient (*D*), mobility (*μ*), and the concentration of mobile charge carriers (*n*) were calculated according to the equations [27]:

(2) $$D=\frac{d^{2}\omega_{2}}{\delta^{2}}$$

where *d* is half of the electrolyte layer thickness, *δ* is the ratio of squares of circular frequencies: ${\omega_{1}}^{2}/{\omega_{2}}^{2}$, where *ω*_1_ is the frequency at Z″(max), *ω*_2_ is the frequency at Z″(min).

(3) $$\mu =\frac{eD}{kT}\;$$

where 2*d* is the electrolyte layer thickness, *S* is the cross-section area of the electrolyte, *R* is the resistance of the electrolyte.

The activation energy of electrolytes was determined graphically, by measuring specific resistivity in the temperature range of 20–50 °C and determining the slope of the line in the coordinate plane with the ordinates. ln *ρ* and abscissae 1/*T*.

The transport number of lithium ions ($t_{Li}^{+}$) was obtained according to the Evans method [28], by polarization of a symmetrical cell Li/GPE/Li with direct current (DC) in combination with the method of impedance spectroscopy and subsequent calculation by the equation:

(4) $$t_{Li}^{+}=\frac{I_{s}\left(\Delta V-R_{0}I_{0}\right)}{I_{0}\left(\Delta V-R_{s}I_{s}\right)}$$

where *I*_0_ and *I*_s_ are the initial and stationary values of direct current, respectively; *R*_0_ and *R*_s_ are the initial and stationary values of volume resistance, respectively; and Δ*V* is the applied potential.

## 3. Results and Discussion

**Figure 1:**
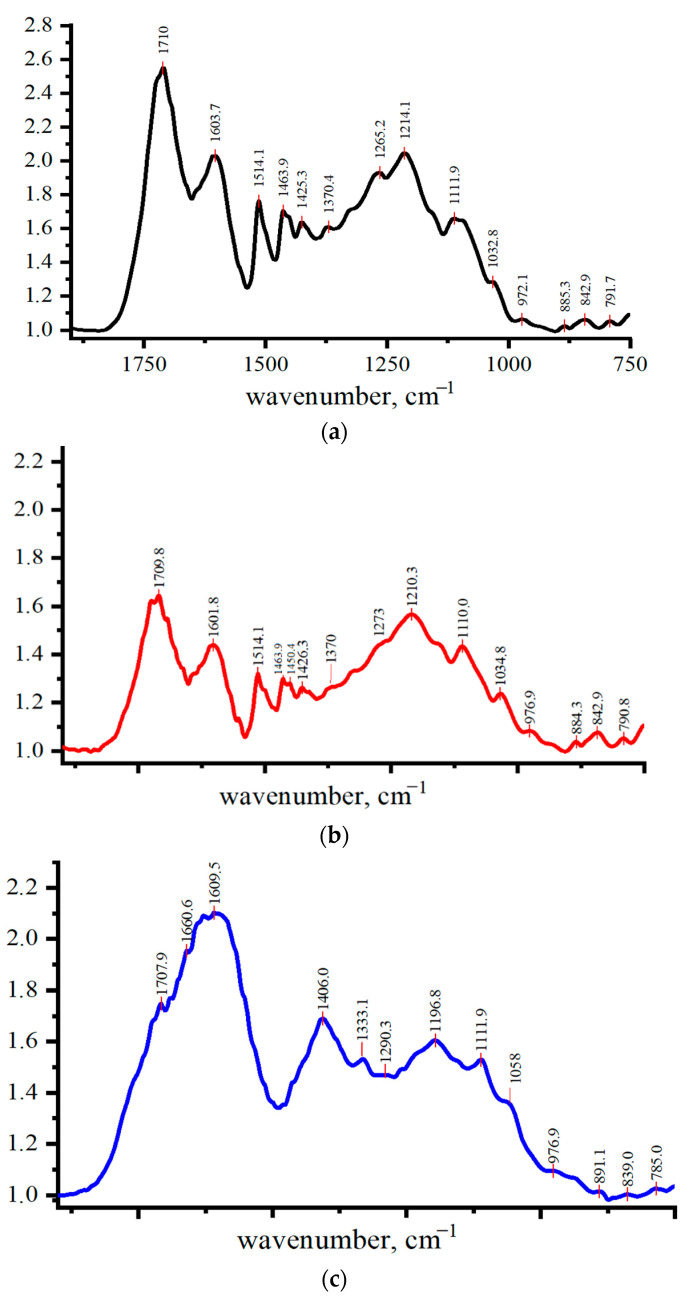
IR spectra of lignin (L) (**a**), lignosulfonate (LS) (**b**), and chloro-lignosulfonate (LS-Cl) (**c**) within the frequency range of 750–1800 cm^−1^.

The results of chemical modification of L were studied by IR spectrometer in the spectral range of 1800–700 cm^−1^, where the greatest number of absorption bands of the samples were observed (Figure 1, Table 1). This area is known as the “fingerprint” region, which is suitable for identifying complex compounds such as L and determining the nature of their structural changes.

The experimental frequencies of the IR absorption bands of main vibrations in the range of 1800–700 cm^−^^1^ are presented in the table, where their possible interpretation is given with allowance for the literature data [29,30,31,32,33,34,35,36,37,38].

Based on the results of IR spectroscopy, chemical transformations occurring during the process of L modification under various conditions can be tracked. The processes occurring in the solutions during L chemical modification have been studied in sufficient details by various authors [14,31,34,35,36,37,38,39,40,41,42,43,44].

As can be seen from Figure 1a, a characteristic feature of the initial L is a high intensity of the absorption line of 1710 cm^−1^, corresponding to stretching vibrations of carbonyl and carboxyl groups as well as the intense line of 1214 cm^−1^ assigned to the OH group conjugated to the aromatic ring (Ar-OH).

A detailed description of the possible transformations of lignin as a result of chemical exposure is given in Supplementary Material Figure S3.

**Figure 2:**
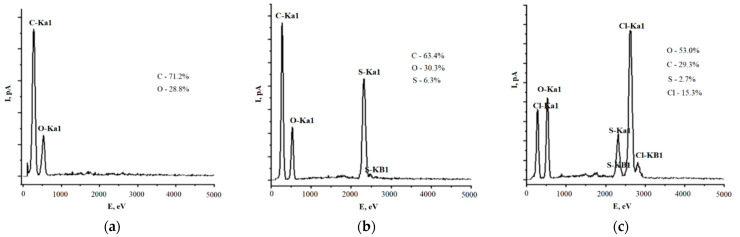
Elemental EDX analysis of the samples: (**a**) L; (**b**) LS; (**c**) LS-Cl.

The resulting L compounds were investigated using an EDX to determine the elemental composition of samples. The elemental analysis of L revealed the degree of incorporation of chlorine and sulfur into L macromolecule (Figure 2).

As can be seen from the figure, the initial L consists of 71.2 wt.% of carbon and 28.8 wt.% of oxygen. In the process of sulfonation, 6.3 wt.% of sulfur was bound with L molecules, which corresponds to one sulfo group per three phenylpropane units. Besides, there is a slight increase in the oxygen content with respect to carbon.

The high reactivity of active chlorine led to its more substantial penetration into the polymer matrix, the values of which reached 15.3% of the total identified mass of the sample. This content corresponds to about 5 chlorine atoms per three phenylpropane units. However, high oxygen content in LS-Cl should also be mentioned, which is probably due to oxidative processes taking place with the participation of oxygen formed during the reaction of hypochlorite with L macromolecule. In addition, high values of oxygen in the spectrum are possibly due to the content of bound water, which is also consistent with the results of IR spectroscopy.

To determine the amount of lithium required to neutralize the acid groups of L, the samples were titrated with 0.1 M of LiOH solution. As a result of titration and subsequent calculation, the following values were determined: 0.0055, 0.0072, and 0.0077 mol/g for the samples L, LS, and LS-Cl, respectively. In the original L, lithium absorption is mainly due to neutralization of acid phenolic hydroxyls.

Sulfonation of L leads to 30% increase in the absorption of Li ions, which is obviously associated with neutralization of the sulfo group. Chlorination of L also leads to an increase of this parameter, but the increase is about 7%, which is probably due to the formation of complexes with coordination bonds between chlorine and lithium introduced into the polymer structure.

**Figure 3:**
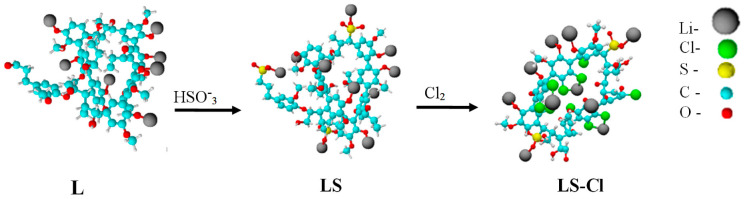
Fragments of L, LS, and LS-Cl molecules.

Thus, taking into account general ideas of the structure of L, the most probable chemical transformations, as well as the elemental composition of the samples obtained by EDX analysis and titration with lithium hydroxide was taken into account, fragments of L macromolecules were simulated for further quantum-chemical calculation of electronic and geometric parameters in order to determine the effect of chemical modification on the polarity of Li atoms conjugated to various functional groups of the L (Figure 3). More detailed information on the structure and types of chemical bonds can be found on the schematic 2D image of the molecules shown in Figure S3.

The models shown in the figure have the following empirical formulas: L–C_80_H_83_Li_7_O_26_; LS–C_80_H_80_Li_10_O_31_S_3_; LS-C–lC_59_H_37_Cl_10_Li_10_O_26_ S_2_.

Although the methods of computational chemistry have a number of restrictions associated with the fact that the models do not take into account a number of parameters, including the actual size of the molecules, their environment, as well as the peculiarity of the supramolecular structure of the polymer [45,46]. However, the results of quantum-chemical calculations can be useful in interpreting the mechanism of charge transfer and establishing the effect of chemical modification of L on its electrical properties.

The charges on lithium atoms calculated by the semi-empirical PM3 method and the geometric characteristics of the bonds of Li atoms with the functional groups of L, LS, and LS-Cl compounds are given in Table 2.

The table presents average values of the characteristic charges of lithium (*q* (Li)) and associated atoms (*q* (A*)), as well as the length of bonds (*l* (Li–A*)) and the total dipole moment (μ) of the fragment of the L macromolecule. The calculation shows that in the pristine L, the bonds of lithium with phenolic hydroxyl Ar–O–Li have a certain polarity due to the shift of electron density from lithium to more electronegative oxygen. Incorporation of sulfo groups into the L macromolecule leads to a noticeable increase in polarity of this bond due to redistribution of electron density. In this case, the R–SO_2_–O–Li bond has the highest polarity in the L molecules. This may testify an increased solvating ability of lithium atoms in the environment of polar solvents.

In the LS-Cl compound, polarity of the Ar–O–Li, R–SO_2_–O–Li bonds significantly increases due to incorporation of chlorine atoms into phenolic units of L. At the same time, in coordination bond with lithium (Ar–Cl–Li), chlorine has a positive charge, most likely due to the positive mesomeric effect in the benzene nucleus. In addition, the total dipole moments of fragments of model the L macromolecules increase with an increase in the degree of its modification and reaches the highest value in the model compound LS-Cl.

Thus, it should be noted that sequential sulfonation and chlorination of the L leads to a noticeable polarization of lithium atoms and the whole molecule. As is known, an increase in polarity of molecules results in an increase of their solvating ability in polar solvents [46,47], which in turn has a significant effect on electrical characteristics of ionic systems.

To study the electrical properties of GPEs, we used the method of impedance spectroscopy.

**Figure 4:**
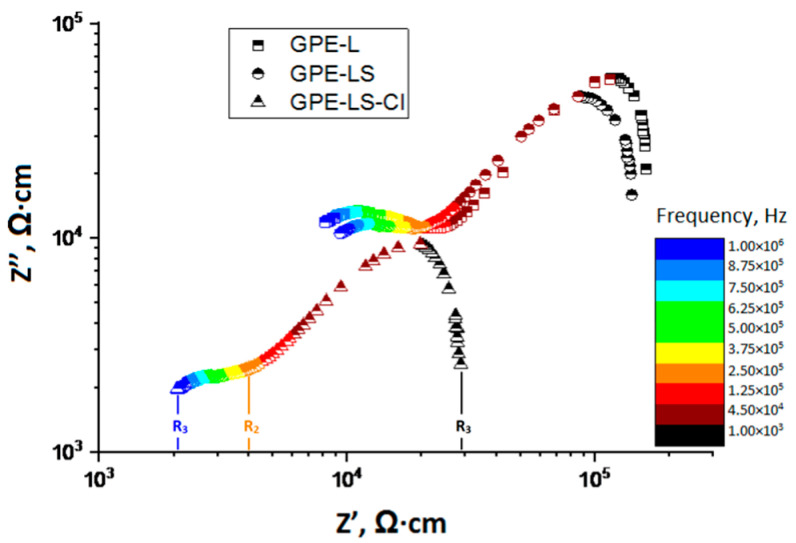
Hodograph of the impedance of the gel polymer electrolytes (GPEs) based on low-molecular-weight L (GPE-L), lignosulfonate (GPE-LS), and chloro-lignosulfonate (GPE-LS-Cl), at 25° C.

Figure 4 shows the impedance spectra for the Li/GPE/Li cell obtained at frequencies of 25 Hz -1 MHz and the signal amplitude of 100 mV at 25 °C in a logarithmic scale. Data presentation in a logarithmic scale is very convenient because it allows presenting various dependencies with a wide range of values on the same coordinate plane.

As can be seen from the figure, the hodographs of GPE-L, GPE-LS, GPE-LS-Cl samples are represented by two characteristic sections in the low-frequency (right) and high-frequency (left) regions limited by the corresponding minima (R_1_, R_2_, R_3_), as shown by the example of GPE-LS-Cl spectrum.

It should be assumed that the low-frequency region is the region that characterizes the processes occurring at the electrode–electrolyte interface. The corresponding values of the R_1_ minima in this region reflects the process of mass transfer of lithium ions between the electrolyte and the electrode. While decreasing frequency, we observe an increase in the reactive component of the impedance, due to the process of charge accumulation at the electrode–electrolyte interface, which is possible when a passivating hydroxide layer forms on the lithium surface. When frequencies are reached, with a *R*_2_ minimum, mass transfer processes prevail in the electrolyte volume, i.e., this value is the volume resistance of the electrolyte. However, the volume of the GPE is not a homogeneous medium and consists of macromolecules combined into supramolecular structures [48,49], which in turn are combined into a common polymer matrix. Besides, the liquid phase in the electrolyte composition can both have a plasticizing effect on the polymer and be present in it as a separate phase [50]. Thus, the difference in electrical properties of the structural elements of the electrolyte is reflected in the graph by the presence of a semicircle in the high-frequency region of the impedance with the finite resistance *R*_3_.

The calculation data of the electrical parameters-*σ*, *D*, *μ*, n for the samples are shown in Table 3. When comparing the obtained results, it should be noted that the conductivity value of the GPE-L sample is low, about 4.19 × 10^−5^ S cm^−1^. Sulfonation of L leads to only a slight (about 20%) increase in conductivity of GPE-LS, still low for practical uses. Additional chlorination of the LS leads to a more noticeable increase in conductivity of GPE-LS-Cl, which is about 2.48 × 10^−4^, and is comparable with the conductivity of most polymer electrolytes [1,2,21]. As can be seen from the table, the increase in conductivity of the samples is mainly due to an increase in the concentration of charge carriers, which, based on a quantum chemical calculation, should be attributed to an increase in the polarity of fragments of the LS-Cl macromolecule and, as a consequence, an increase in the solvating ability of lithium ions in it. At the same time, the diffusion coefficient and mobility of charge carriers noticeably decrease depending on the degree of the L modification, which may be related to the structural features of the Ls in GPEs.

Thus, based on the results of the research, we can assume that modification influences the structural and electrical properties of L. As noted above, the results of quantum chemical calculations show that sulfonation and then chlorination of L lead to a noticeable polarization of lithium atoms and the whole molecule. These calculations agree quite well with the results of electronic and thermal studies (S4), where such polarization can cause an increase in charge carriers, as well as the ability of the polymer to retain the liquid phase, due to the more active interaction of fragments of the L macromolecule with a polar solvent DMSO.

**Figure 5:**
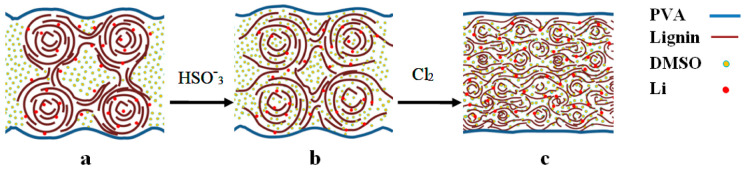
The structure of the gel polymer electrolytes: (**a**)-GPE-L;(**b**)-GPE-LS; (**c**)-GPE-LS-Cl.

Figure 5 presents an image of the electrolyte micro regions containing various types of the L. As can be seen from Figure 5a, the GPE consists mainly of dense clusters, interconnected by polymer bridges [45,48,49,51]. The volume between these clusters is filled with liquid electrolyte and forms a heterogeneous system of solid and liquid phases. Consequently, in the GPE with the initial L, most of the lithium ions are blocked in the bulk of such structures, and cations located at the polymer-DMSO interface are mainly involved in charge transfer. For this reason, high values of mobility and diffusion coefficient are recorded, since surface cations have a greater degree of freedom and are less associated with the polymer matrix. Besides, the low conductivity value may be due to the fact that the internal volume of the clusters acts as a dielectric, reducing the total conductivity of the GPE volume.

The results of quantum chemical calculations show that the sulfonated L has a higher polarity of macromolecule fragments than its precursor, which in turn increases its solvating ability, leading to the penetration of DMSO into the volume of polymer clusters and their swelling (Figure 5b). In this case, the volume of free solvent decreases markedly, as does the heterogeneity of the system as a whole; as a result there is a noticeable decrease in the values of the diffusion coefficient and mobility of charge carriers. On the other hand, this causes an additional increase in the concentration of mobile charge carriers and the release of some of them for the charge transfer process in the electrolyte volume.

Due to its high polarity, chloro-lignosulfonate reacts most actively with the solvent, forming clusters that are much smaller than its precursors. It results in the formation of an almost homogeneous medium with a small gradient of polymer density in the electrolyte volume (Figure 5a). And almost all of the solvent is in a bound state. Such structural transformations lead to a noticeable increase in the concentration of charge carriers, on the one hand, and a decrease in the volume resistance of the electrolyte, on the other hand, which causes a sharp increase in the conductivity of the electrolyte based on LS-Cl.

**Figure 6:**
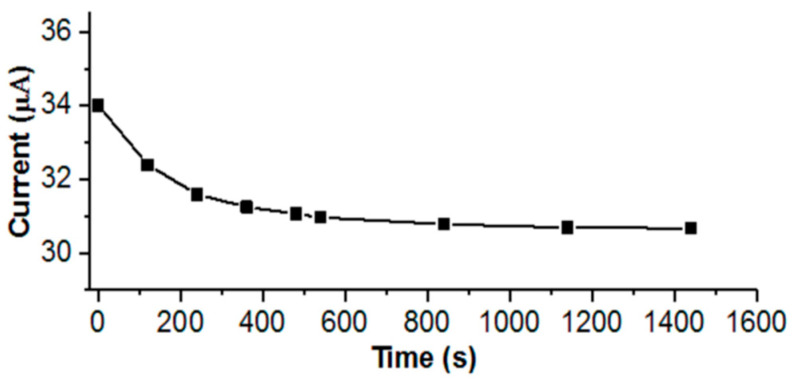
Dependence of displacement current on time.

Thus, sequential sulfonation and chlorination of L allows one to obtain an advanced material for the formation of the GPE with electrical conductivity suitable for its practical use GPE-LS-Cl. Therefore, additional research was carried out for him to establish the transfer number of the lithium cation (Figure 6). The transport number of lithium ions $t_{Li}^{+}$ is one of the most important parameters of electrolytes that determine the ability of charge and discharge of LIB. Measuring $t_{Li\;}^{+}$ by the method proposed by Evans et al. [28] it was determined that the transport number of lithium ions is 0.89 at 50 °C (Figure 6, Table 4).

The obtained value is the highest among those available in the literature to date. For GPEs based on traditional polymer matrices, the range of transport numbers of lithium ions is usually from 0.20 to 0.70 [52,53], reaching, in some cases, the values of 0.85 0.87 [18,52]. However, the commercial separator Celgard 2730 provides only $t_{Li}^{+}$ 0.27 [54].

## 4. Conclusions

This paper showed that LS-Cl-based GPE, that have a high transport number of lithium ions and acceptable conductivity values, can be one of the advanced materials for LIBs, which can provide fast charging and discharging of the batteries, and high current density. In addition, the use of lignin meets not only technological requirements, but also ensures environmental friendliness of the materials based on it. Moreover, this breaks new ground in using large-tonnage waste, which can provide cheap and available raw materials for production of products with high added value.

## Figures and Tables

**Table 1:** Absorption bands of lignin base oscillation in the 1800–700 cm^−1^ range.

Wavenumber, cm^−1^	Interpretation of Absorption Bands
1710	Stretching vibrations of the R-C=O bond in non-conjugated ketones of carbonyl, carboxyl, and ether groups
1660	Oscillations of the Ar=O bond conjugated to the aromatic ring
1610, 1615, 1515, 1423	Skeletal vibrations of the aromatic ring
1457	Deformation vibrations O-CH_3_; scissor vibrations CH_2_
1374	Deformation vibrations of the C-H bond in C-CH_3_; deformation plane vibrations of phenolic O-H groups.
1325	Vibrations of the syringyl ring with stretching vibrations of CO groups.
1265	Vibrations of the guaiacyl ring with stretching vibrations of CO groups.
1215	Ar-Ovibrations in Ar-OH and Ar-O-CH_3_
1152	Plane deformation vibrations of aromatic CH bonds
1032	Plane deformation vibrations of CH; stretching vibrations of SO_3_ groups
972	Out-of-plane deformation vibrations of the CCH groups in trans-HC=CH

**Table 2:** Electronic and geometric characteristics of the bonds of Li atoms with functional groups calculated by the PM3 method.

Sample	Bond of Li Atom with Functional Group	*q* (Li)	*q* (A *)	*l* (Li–A *), Å	μ, D
L	Ar–O–Li	0.421	−0.436	1.773	11.677
LS	Ar–O–Li	0.452	−0.463	1.753	18.192
	R–SO2–O–Li	0.480	−0.814	1.773	
LS-Cl	Ar–O–Li	0.544	−0.412	1.862	25.981
	R–SO2–O–Li	0.653	−0.832	1.766	
	Ar–Cl–Li	0.396	0.232	2.321	

* Charge of anion atom directly associated with lithium ion.

**Table 3:** Electrical parameters of the GPS.

Electrolyte	Electrical Parameters			
	*σ*, *S* cm^−1^	*D*, cm^2^s^−1^	*μ*, cm^2^V^−1^s^−1^	*n*, cm^−3^
GPE-L	4.19 × 10^−5^	8.64 × 10^−5^	3.37 × 10^−3^	7.76 × 10^+16^
GPE-LS	5.01 × 10^−5^	3.70 × 10^−7^	1.44 × 10^−5^	2.17 × 10^+19^
GPE-LS-Cl	2.48 × 10^−4^	3.06 × 10^−7^	1.19 × 10^−5^	1.30 × 10^+20^

**Table 4:** The values of the initial and steady current, the initial and steady resistance, and the transport number of lithium ions.

Electrolyte	*V*, mV	*I*s, μA	*I*o, μA	*R*s, Ohm	*R*o, Ohm	$t_{Li}^{+}$
GPE-LS-Cl	20	30.6	34.0	711.5	693.5	0.89

## Data Availability

Research data are available upon reasonable request.

## Supplementary Materials

The following are available online at https://www.mdpi.com/article/10.3390/polym13142306/s1, Figure S1: Gel polymer electrolyte based on low-molecular-weight lignin (GPE-L), lignosulfonate (GPE-LS) and chloro-lignosulfonate (GPE-LS-Cl). Figure S2: Electrochemical cell with a clamping electrode: (a) model, internal structure; (b) photo of a cell on a water heater; Figure S3: information on the structure and types of chemical bonds; Figure S4: results of electronic and thermal studies.
