# Supplementary material for: Lignin-Based Gel Polymer Electrolyte for Cationic Conductivity
Source: Polymers (Basel). 2021 Jul 14;13(14):2306. doi: 10.3390/polym13142306 (PMC8309217; doi:10.3390/polym13142306)
Supplement: Supplementary file 1 [file polymers-13-02306-s001.zip › polymers-1300435-supplementary.pdf]

Lignin-based Gel Polymer Electrolyte for Cationic Conductivity

Nabi S. Shabanov, Kamil Sh. Rabadanov, Malik M. Gafurov, Abdulgalim B. Isaev, Dinara S. Sobola, Sagim I. Suleimanov, Akhmed M Amirov, Abil Sh. Asvarov.

S1

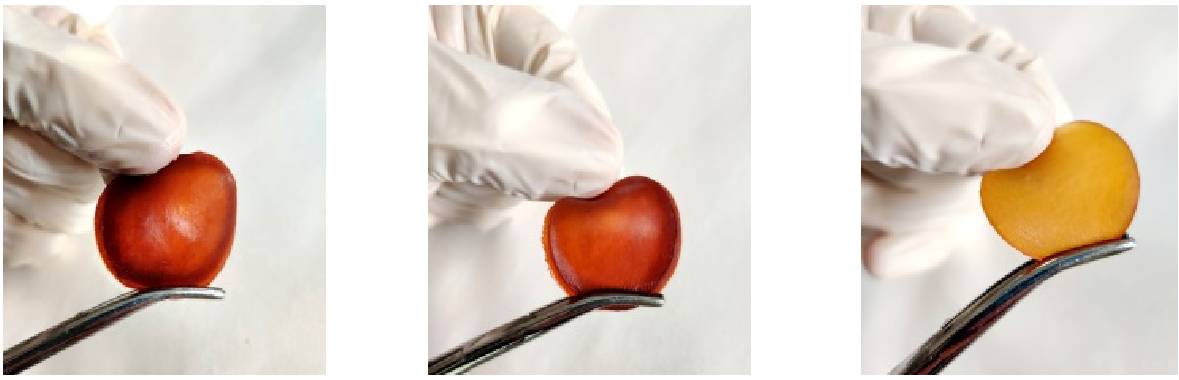

(GPE-L) (GPE-LS) (GPE-LS-Cl)

Gel polymer electrolyte based on low-molecular-weight lignin (GPE-L), lignosulfonate (GPE-LS) and chloro-lignosulfonate (GPE-LS-Cl).

S2

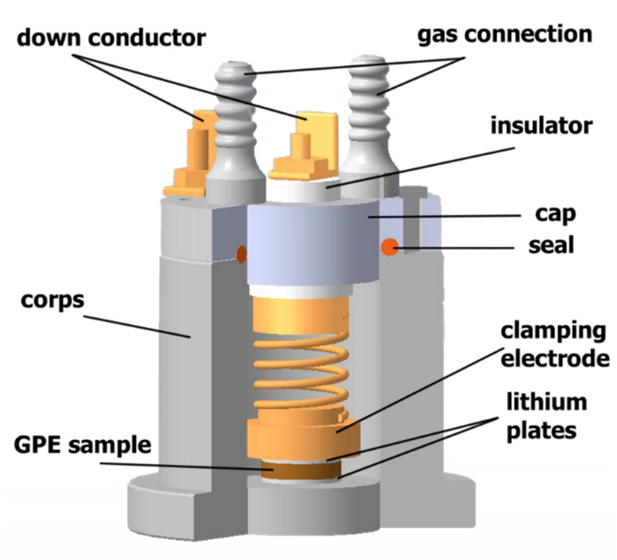

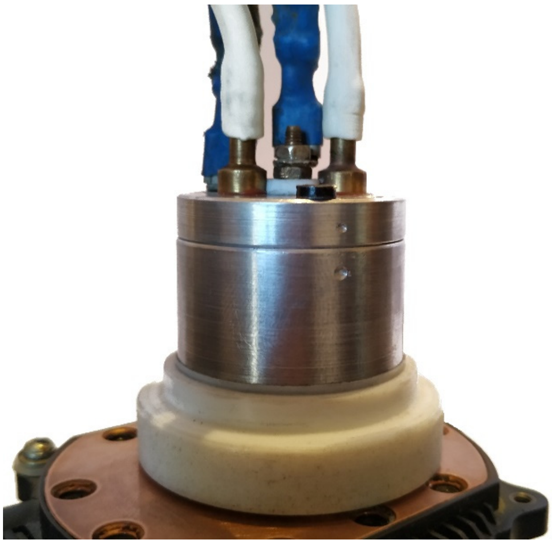

(a) (b)

Electrochemical cell with a clamping electrode: (a) model, internal structure; (b) photo of a cell on a water heater

S3: Structure and types of chemical bonds

Thus, during alkaline hydrolysis of the L, the reactions of breaking of alkyl–aryl ether bonds mainly take place, which are accompanied by fragmentation of the L macromolecule and its transition into the solution in the form of sodium phenolates.

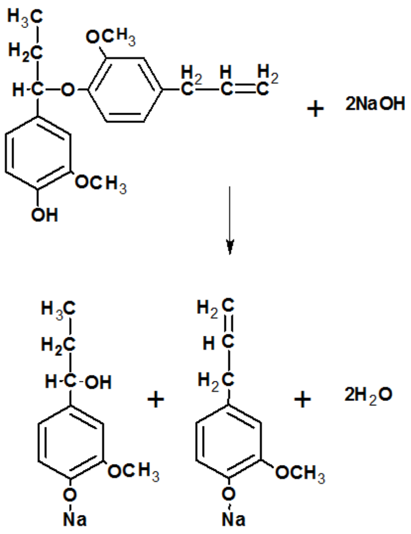

(1)

After sulfonation (Fig. 1b), the structure of the initial L did not change significantly, except for an increase in absorption in the region of 1034 cm^{-1}, which is caused by overlapping of vibration lines of SO_3 groups.

Benzyl alcohol residues or their ethers in phenolic and non-phenolic units take part in sulfonation reactions. The mechanism of sulfonation reaction will be determined by the laws of the reaction of nucleophilic substitution in a neutral medium, in which phenolic units are predominantly sulfonated.

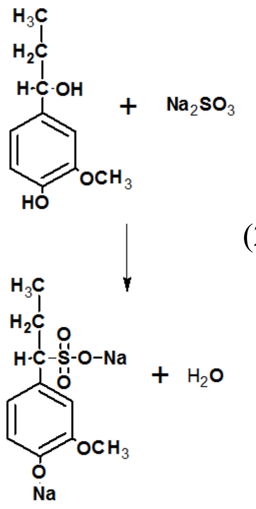

(2)

In addition, aldehyde and ketone carbonyl groups are sulfonated (R–C=O). The reaction of carbonyl groups can proceed through the reversible formation of fenols followed by the reaction of the hydroxyl group with sodium sulfite.

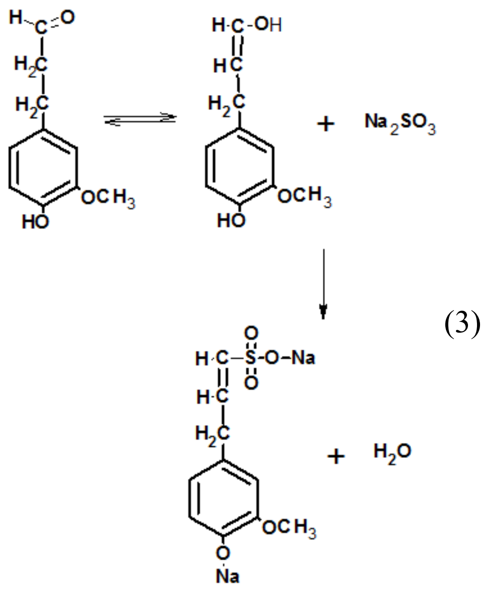

(3)

In the IR spectra, this reaction is reflected in a decrease in the line intensity at 1710 cm^{-1}, which is characteristic of the aldehyde and ketone R–C=O groups, and an increase in line intensity at 972 cm^{-1} with a slight shift to the high-frequency region. It should be noted that there is no shift in the frequencies of vibrations and changes in the intensities of the absorption bands of skeletal vibrations of the benzene ring, which allows us to assume the absence of any effect of sulfo-groups on the aromatic structure.

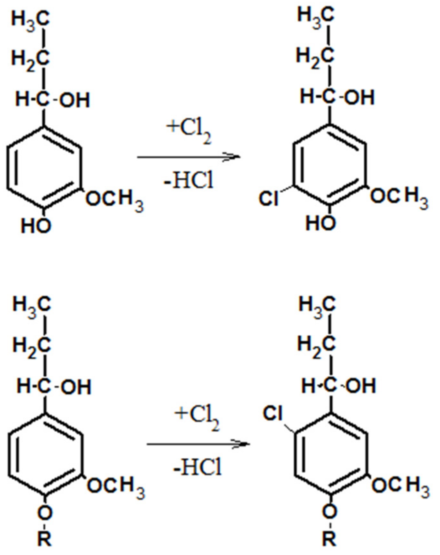

(4)

The most significant changes are observed in the IR absorption spectrum after chlorination of the L (Fig. 1c). Characteristic differences include the shift of the vibrational peaks of the benzene ring due to electrophilic substitution of hydrogen atoms by heavier chlorine atoms. This reaction proceeds in phenolic units mainly in the 5th position, and in non-phenolic units mainly in the 6th position.

A new absorption peak is recorded at 1660 cm^{-1}, which is assigned to the vibration band of Ar=O groups conjugated to the aromatic ring. Since the reaction proceeds in an aqueous medium, the

appearance of these bonds is possible during demethylation of the benzene ring by oxygen until the formation of ortho-quinones.

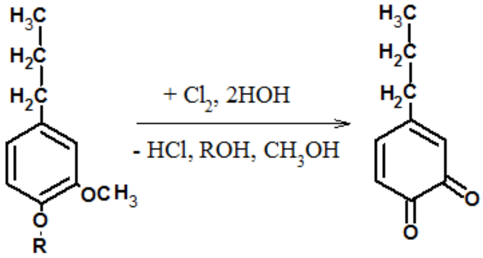

(5)

It should be noted that the intensity within the region of 1700–1600 cm^{-1} can also be affected by bound water (1635 cm^{-1} for bending vibration of water).

An increase in the absorption of the lines of skeletal vibrations of the aromatic ring (1601 cm^{-1} and 1405 cm^{-1}) and the bonds conjugated to it, against the background of all other peaks, may be associated with an increase in the phenolic component in the total mass of the L due to detaching of the propane chain on account of deeper chlorination. The reaction proceeds more easily in phenolic units than in non-phenolic ones. The presence of a hydroxyl or carboxyl group at the α-position contributes to this reaction.

Then, during the process of filtration, the water-soluble product of the reaction—propanal aldehyde or propanoic acid—leaks out of the total mass of the L, which leads to an increase in the concentration of its phenolic component.

Thus, with a reasonable degree of certainty, we can assume the main direction of chemical transformations. There is no doubt that these reactions cannot fully reflect a wide range of the L transformations. However, spectral studies and experience allow us to determine the main directions of chemical reactions that occur during L modification.

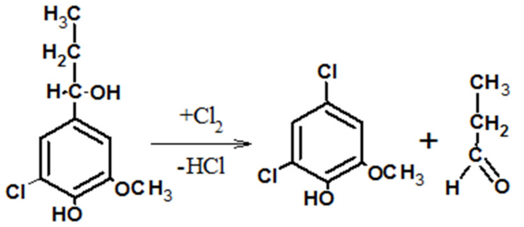

(6)

or

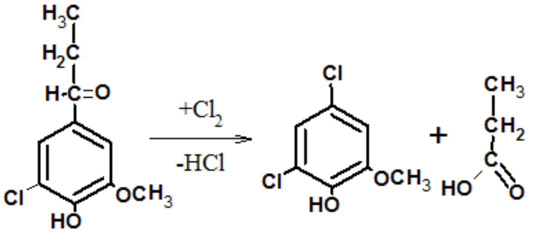

(7)

S4: Electronic and thermal studies

Model L is a fragment consisting of 8 phenylpropane units, in which hydrogen atoms in the phenolic hydroxyl are replaced by lithium atoms (Ar–O–Li).

As a result of sulfonation, the structure turns into the LS model. The LS structure retains the number of phenylpropane units, while 1 carbonyl and 2 alcohol groups are replaced by the corresponding number of sulfogroups (reaction schemes 2,3). This model has two types of lithium, one of them replaces the phenolic hydrogen–Ar–O–Li, and the other replaces the hydrogen of sulfonic acid ($R–SO_2–O–Li$). The third LS-Cl model is a fragment of lithium chloro-ligninosulfonate macromolecule. Since L undergoes intense degradation during chlorination, this model is based on 7 phenylpropane units, three of which do not have a propane unit (reaction schemes 6, 7), and the number of sulfo groups is reduced to two. Two phenolic units turned into ortho-quinone (reaction scheme 5). Chlorine is localized in phenolic units according to reaction schemes 4, 6, 7. Since it is assumed that the increase in lithium saturation in LS-Cl is associated with the formation of a complex compound (Fig. 3), the LS-Cl model has the third type of lithium, which forms a coordination bond with phenolic chlorine (Ar–Cl---Li).

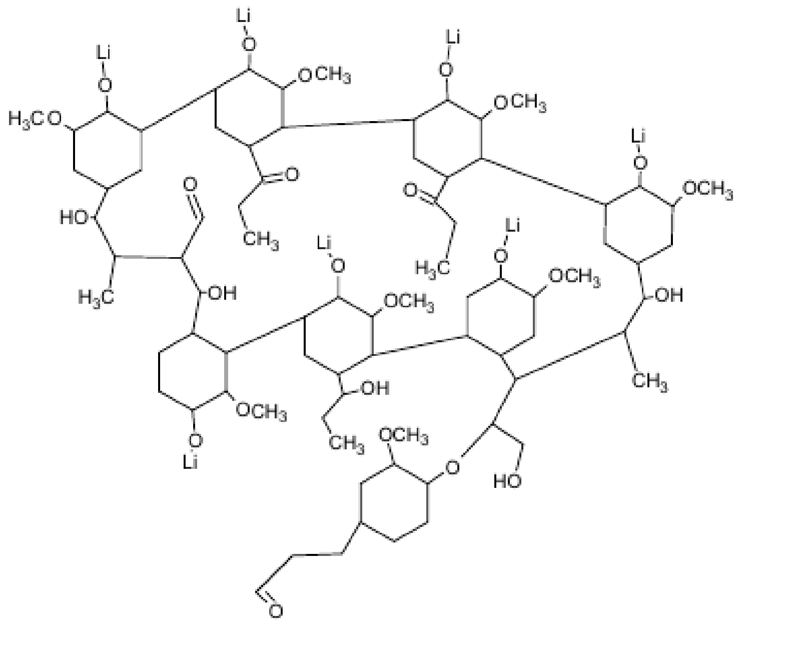	L - fragment of the original lignin

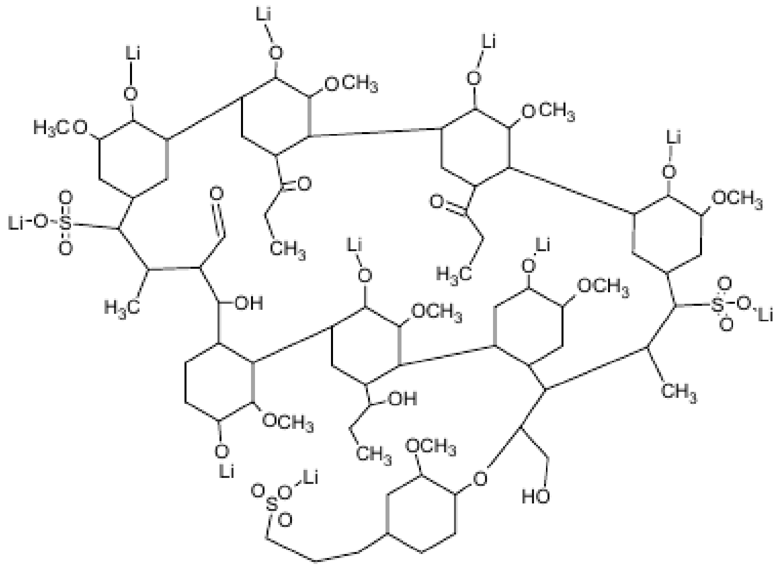	LS - fragment of sulfonated lignin
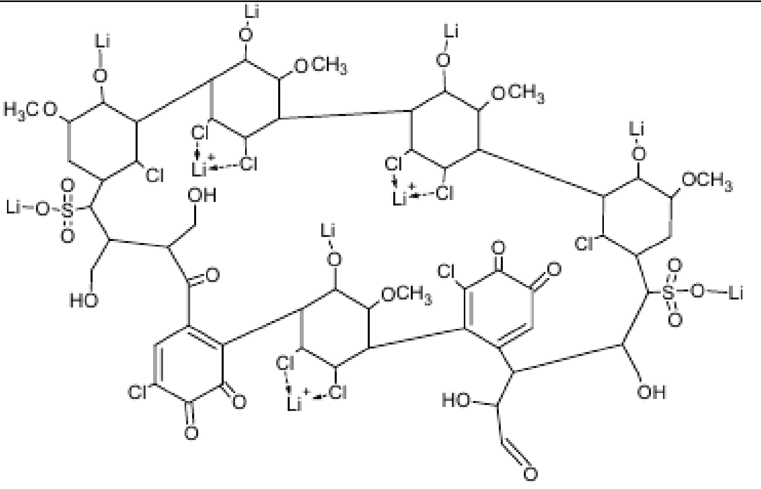	LS-Cl fragment of chlorinated sulfolignin

S4

The GPEs obtained on the basis of modified Ls were studied by the methods of thermogravimetric analysis (TGA) and differential thermal analysis (DTA). These methods make it possible to determine the content of the liquid phase in the composition of the samples, which is the main indicator on which ion conductivity in electrolytes of this type depends.

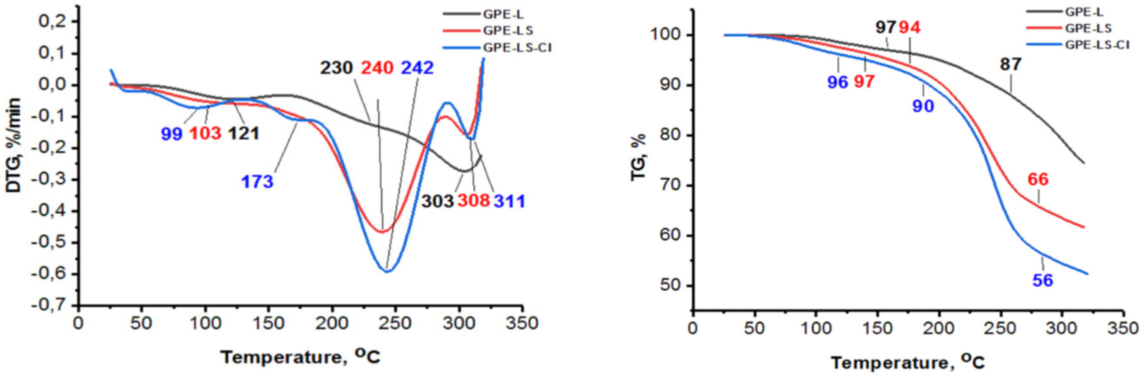

a) b)

Figure 4. Differential thermal analysis: a) and thermogravimetric analysis b) of the samples GPE-L; GPE-LS; GPE-LS-Cl.

As can be seen from figure, DTA analysis makes it possible to separate the processes that occur during heating of electrolytes into four successive stages, which are identified as: 1—evaporation of free water, 2—evaporation of bound water, 3—evaporation of DMSO and 4—decomposition of the L. The first two processes on the DTA curves of GPE-L are combined in one peak with a broad base stretching from 75 to 153 °C. With the increase of temperature, a weak peak is observed, which is attributed to the evaporation of DMSO, with a maximum rate at 230 °C, and the subsequent decomposition of L at 303 °C. The analysis curve of GPE-LS is characterized by stronger peaks related to the evaporation of DMSO and the decomposition of the L, while the temperatures of these processes shift by +10 and + 5 ° C, respectively. The process of free water evaporation in GPE-LS is observed in the temperature range of 66–139 °C, while the region, which can be attributed to the evaporation of bound water, in the temperature range of 140–175 °C, is weakly expressed. In the GPE-LS-Cl electrolyte, the process of evaporation of free and bound water is clearly visible and resolved by temperature. In total, these processes take place within the range from 60 to 182 °C with characteristic minima on the curves. The peak characterizing the process of DMSO evaporation is more pronounced, with a shift in the temperature of the intensive phase by + 12 °C as compared to GPE-L and +2 °C as compared to GPE-SL. A shift in the

temperature of the liquid phase evaporation to higher temperatures indicates the formation of a thermodynamically stronger bond of L with the solvent.

The amount of retained liquid in the volume of the polymer electrolyte could be estimated using TG analysis, which presents the values of the relative mass of the samples at the points corresponding to the end of the above processes.

Thus, modification of the L leads to a noticeable increase in the content of the liquid phase in the electrolyte volume. As compared to GPE-L, in electrolytes with modified L, the amount of the liquid phase increased by 3.5 times for GPE-SL and by 5.4 times for GPE-LS-Cl. GPE-LS-Cl is characterized by a noticeable increase in the proportion of bound water, which is retained in the volume of the polymer electrolyte up to 182 °C, probably due to the influence of the increased polarity of the L macromolecule fragments.
